# Migrated Tubal Ligation (Filshie) Clip as an Uncommon Cause of Chronic Abdominal Pain

**DOI:** 10.1155/2020/4809859

**Published:** 2020-02-10

**Authors:** Sahil Sharma, Radek Martyniak, Vladislav Khokhotva

**Affiliations:** Schulich School of Medicine, Western University, Canada

## Abstract

Tubal ligation (TL) is an effective and common method of fertility control. In the year 2009, over 24,000 were performed in Canada alone. Migration of Filshie clips used during TL is estimated to occur in 25% of all patients; 0.1-0.6% of these patients subsequently experience symptoms or extrusion of the clip from anatomical sites such as the anus, vagina, urethra, or abdominal wall. Migrated clips may present as chronic groin sinus, perianal sepsis, or chronic abdominal pain. These symptoms can occur as early as 6 weeks or as late as 21 years after application. We present the case of a 49-year-old female with a 3.5-year history of intermittent dull nonradiating left upper quadrant (LUQ) pain lasting on average 2-3 days. There were no other associated symptoms, and the longest pain-free period was 4 days. Her past medical history includes COPD, GERD, IBS, and depression. Current medications are only remarkable for Symbicort. Pertinent past surgical history includes laparoscopic tubal ligation with Filshie clips in 1999, followed by a vaginal hysterectomy in 2013. Migrated tubal ligation clip was noted on an abdominal X-ray. The patient was then referred for surgical management. Subsequent CT scan confirmed a solitary clip present adjacent to the left lobe of the liver. No other abnormalities were reported. Patient underwent laparoscopy for removal of the clip, which was identified to be underneath the left lobe of the liver embedded in the gastrohepatic omentum. Please see the video link provided. Postoperative pathology report confirmed the presence of a Filshie clip. Patient reported complete resolution of her LUQ pain at a 5-week and 3.5-month follow-up. This case shows that although symptomatic clip migration is a rare phenomenon, it should be given special consideration in women with unexplained chronic abdominal pain and a history of TL. Additionally, removal of clip can provide resolution of symptoms.

## 1. Introduction

Tubal ligation (TL) is a common method of fertility control in North America with over 24,000 performed in the year 2009 in Canada alone [1]. Nearly 85% of these procedures are reported to utilize the Filshie clip as the preferred method of tubal occlusion [2]. Filshie clip is a silicone-lined, titanium, occlusive device which was first introduced for surgical sterilization in 1981 [3]. Migration of Filshie clips after application is a common phenomenon and can result in failed sterilization [4]. Recent literature has also identified migrated Filshie clips to be the source of pain, discomfort, and abscess formation [5]. Though migrated Filshie clips are infrequently symptomatic, their frequency of use in gynecological procedures makes knowledge of potential adverse sequelae clinically relevant [6]. In this report, we present the case of a woman experiencing chronic abdominal pain secondary to a migrated Filshie clip following TL.

## 2. Case Report

A 49-year-old female was referred to the General Surgery Clinic with a 3.5-year history of intermittent left upper quadrant (LUQ) pain. She described her symptoms as a dull localized pain with gradual onset lasting on average 2-3 days. When present, the pain fluctuated in intensity throughout the day. This pain was noncyclic and had no identifiable triggers. There was no history of trauma to the area. The pain was not reproducible with palpation of the area. She was unable to find any provoking or palliative factors or positions. Her longest pain-free period was recalled to be approximately 4 days. There were no other associated symptoms. Prior to 3.5 years ago, the patient never had any episodes of similar symptoms. There was no history of unintentional weight loss, fevers, or night sweats. Upon physical exam, the patient's abdomen had no rebound, guarding, or overlying skin changes.

**Figure 1:**
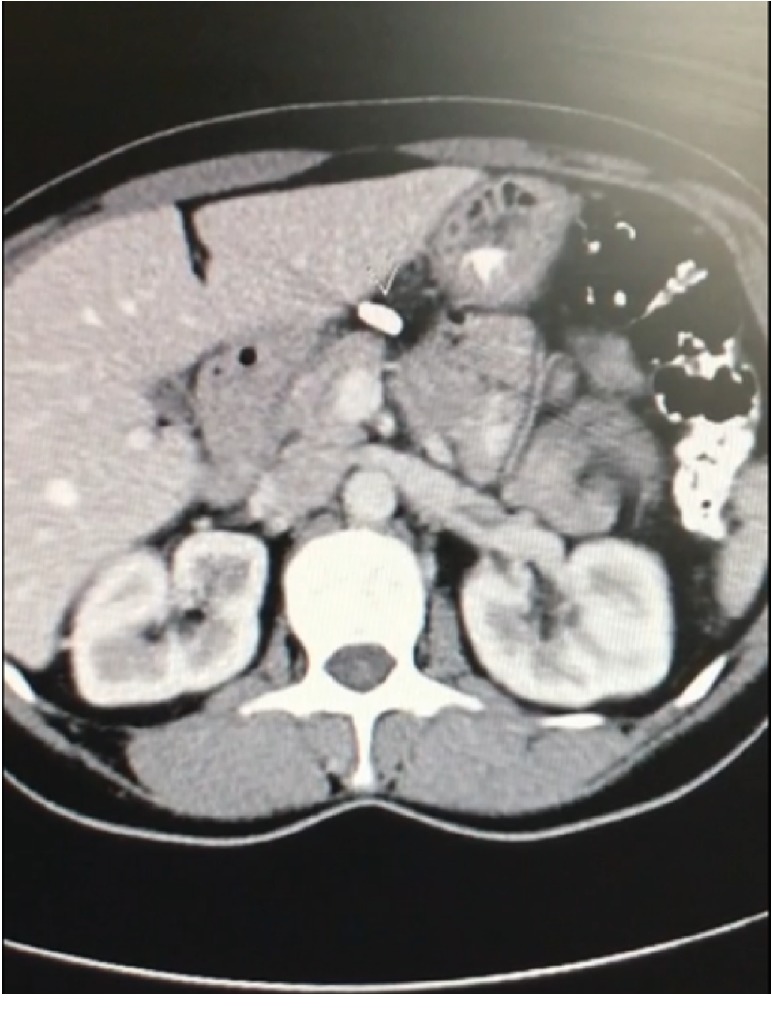
Transverse section of a computed tomography scan of the abdomen showing a migrated Filshie clip lying inferior to the left of the liver.

Prior to the onset of the pain, the patient had undergone an uncomplicated laparoscopic TL with Filshie clips in 1999 and a vaginal hysterectomy in 2013. Initially, the patient's pain was thought to be of gastrointestinal origin for which she underwent a gastroscopy and colonoscopy—both revealing no pathology. The patient was involved in a motor vehicle accident one year prior to presentation at our clinic and during workup of symptoms related to the collision—an incidental finding of one migrated tubal ligation clip was made on plain film. The clip was noted to be present underneath the diaphragm adjacent to the stomach. A subsequent CT scan localized the clip adjacent to the liver, embedded in the gastrohepatic ligament of the liver (Figure 1). There was no other abdominal pathology revealed on imaging.

A diagnostic laparoscopy with the intent of identifying and removing the clip was undertaken. The Filshie clip was seen underneath the left lobe of the liver embedded in the gastrohepatic omentum and was subsequently excised using bipolar diathermy. No other potential causes of her LUQ pain were identified during laparoscopy. The patient was contacted at a 5-week postop and again 3.5 months later and found to be completely symptom-free.

## 3. Discussion

TL is an effective and common method of fertility control [7]. In the year 2009, over 24,000 were performed in Canada alone [1]. Filshie clips are silicone-lined, titanium devices that have been routinely used for sterilization procedures for several decades dating back to 1981 [3]. Complications with Filshie clips can be categorized by those related to the surgery and those related to the clip itself. Complications related to surgery are often related to misapplication of the clip which can lead to future pregnancies. Complications related to the clip are more varied and often present as pain or abscess formation secondary to extrusion or migration of the clip [2].

Migration of Filshie clips used during TL is estimated to occur in 25% of all patients; 0.1-0.6% of these patient subsequently experience symptoms or extrusion of the clip from anatomical sites such as the anus, vagina, urethra, or abdominal wall [5, 8–11]. Migrated clips may present as chronic groin sinus, perianal sepsis, or chronic abdominal pain [12–14]. These symptoms can occur as early as 6 weeks or as late as 21 years after application [12, 15].

**Figure 2:**
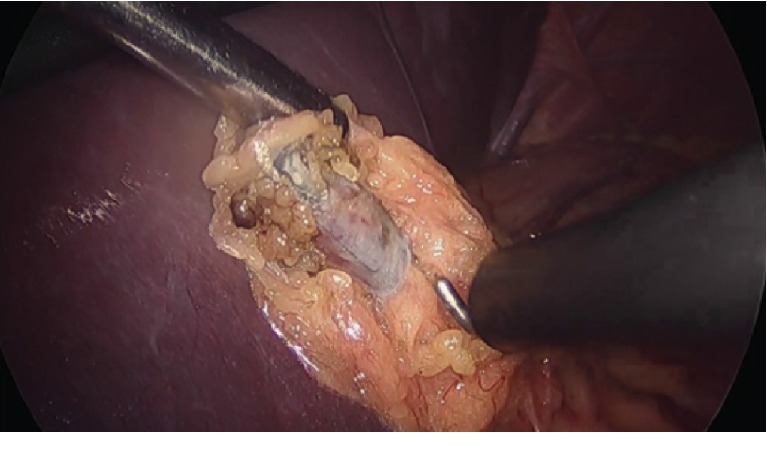
Laparoscopic image of an inflammatory capsule surrounding the migrated Filshie clip during dissection.

The mechanism of Filshie clip migration is thought to be closely related to its mechanism of function. Filshie clip application occludes the nearby vascular structures leading to avascular necrosis. Two blind stumps of the previously continuous structure remain after application, with one stump containing the Filshie clip with its jaws closed. It is theorized that peritoneal growth and adhesion formation of the clip-containing stump encloses the Filshie clip preventing migration. If this fails to occur, migration of the Filshie clip may occur [16]. The inflammatory capsule and adhesions observed surrounding the Filshie clip in this case further support the idea of a localized inflammatory tissue reaction (Figure 2) [17].

Our patient's pain had been occurring for several years and causing a great degree of discomfort. She had undergone multiple other investigations revealing no other identifiable sources of her pain. Laparoscopic investigation of the abdominal cavity failed to reveal any other identifiable sources of pain other than the Filshie clip. Furthermore, removal of the Filshie clip resulted in complete resolution of the pain and the longest pain-free period the patient had experienced in several years. The combination of all these factors led us to believe that the source of the patient's pain was the Filshie clip.

There has been an increasing trend towards bilateral salpingectomies over tubal ligation for the purposes of permanent contraception. This is in light of a potential preventative role in the development of ovarian cancer by removing fallopian tubes which may be a source of origin for epithelial ovarian cancers [18]. This trend may be a step in the right direction given the high estimated rate of Filshie clip migration and potential complications that may arise as a result. This case shows that although symptomatic clip migration is a rare phenomenon, it should be given special consideration in women with unexplained chronic abdominal pain and a history of TL. Additionally, removal of clip can provide resolution of symptoms.
